# The role of School-Based Health Centers in adolescent well-being: a call for action

**DOI:** 10.3389/fpubh.2025.1557124

**Published:** 2025-03-03

**Authors:** Fiorella Quiroz-Cárdenas, José Francisco López-Gil

**Affiliations:** ^1^Health and Social Research Center, Universidad de Castilla-La Mancha, Cuenca, Spain; ^2^One Health Research Group, Universidad de Las Américas, Quito, Ecuador

**Keywords:** adolescent health, preventive care, mental health, health promotion, non-communicable diseases, sexual and reproductive health, primary health care

## Introduction

Adolescence represents a critical developmental and discovery period spanning approximately 10–19 years and is characterized by profound physiological, cognitive, and psychological transformations (1, 2). During this transitional period, individuals progressively acquire greater autonomy and develop personalized lifestyle choices with long-term health implications (3). According to the World Health Organization, there are 1.3 billion adolescents worldwide, and a practical approach to reach most of them is through schools since they spend a considerable part of their time there (4–6). The World Health Organization recommends establishing School-Based Health Centers (SBHCs) that are located inside high schools to ensure adolescents' health and wellbeing (7). Therefore, this letter aims to highlight the significance of SBHCs for adolescents, urging researchers and policymakers to conduct comprehensive research and reassess health policy priorities.

## Call for action: providing comprehensive medical care

Health professionals play a strategic role in SBHCs to provide primary care (8) to adolescents during school days or hours, promoting health equity (9). Thus, SBHCs offer primary health care, which signifies the effective management of acute illnesses (e.g., flu, stomachache, or others) and assistance with injuries resulting from accidents (10–12), falls, trauma, or others, along with the availability of medicine prescriptions (11, 13, 14) and the use of medical equipment for appointments and first aid, respectively.

Consequently, physicians can deal with emergencies derived from students' preexisting health conditions, such as an epileptic attack, asthma, or diabetic crisis (11, 14, 15). Moreover, periodic health screenings to assess adolescents' general condition could detect health problems early and treat or refer them to a specialist (14, 16). This primary medical assistance decreases school absenteeism from going to external doctor appointments, hospitalizations, and visits to emergency departments (2, 7).

The prevalence of overweight and obesity in adolescents worldwide is significantly high, with some studies reporting figures exceeding 30%−40% in certain populations (17). This highlights the increasing importance of addressing nutrition-related health risks (17, 18) and the role of SBHCs in promoting healthier lifestyles among adolescents (19, 20). Primary prevention consists of promoting healthy lifestyles (14) associated with nutrition and physical activity in adolescents, which allows them to prevent non-transmissible diseases such as diabetes, hypertension, or obesity later in life (21, 22) and increases their life expectancy. Providing relevant and age-appropriate information (23) reinforces adolescents' decision-making capacity and gives them self-care abilities (2) to introduce positive lifestyle changes that can continue through later stages of life (24).

Some studies have demonstrated that strategies for preventing obesity are cost- effective (25) and reduce body mass index (5, 26, 27), which could increase adolescents' wellbeing. Similarly, SBHCs that promote nutrition and physical activity shape positive eating habits in school settings (26). Likewise, periodic visits to SBHCs and adolescents' vital signs and anthropometric measures highlight the population at risk of acquiring non-communicable diseases, and health professionals can take action to mitigate this risk or provide early diagnoses and monitoring during adolescents' stay at school (14). Counseling healthy lifestyles among adolescents (28) and screening play important roles in their wellbeing.

In addition to benefits related to healthy lifestyle habits or the prevention of chronic non-communicable diseases, SBHCs provide other essential health services. For example, the World Health Organization recommends human papillomavirus (HPV) vaccination for girls aged nine to fourteen. Immunization may be efficiently managed by SBHCs, advancing to inhibit future cervical cancer. Moreover, sexual and reproductive health education, including HIV prevention, is essential in reducing teen pregnancies and sexually transmitted infections (10, 14). Additionally, providing information and screening related to substance consumption, such as smoking, alcohol usage, and illicit drug use, contributes to a decrease in risky behaviors later in life (10). Collectively, these efforts empower adolescents to become healthier and more well-informed, underscoring the value of SBHCs in promoting youth wellbeing.

## Support and indirect benefits

Evidence suggests that adolescents feel at ease in receiving healthcare services within school (29). As appointments are private time between the health professional and the patient, without parents present, adolescents may feel free to ask questions about any health topic in a confident environment without feeling shame. These confidential conversations encourage adolescents to talk about surrounding high-risk behaviors (30). Moreover, physicians can identify mental, emotional, or behavioral disorders, such as anxiety, depression, or attention-deficit/hyperactivity disorders, during consultations, enabling them to offer treatment or connect patients with appropriate services (10). Research indicates that adolescents who undergo thorough, comprehensive assessments report reduced symptoms of depression (30).

Adolescents may prefer schools with SBHCs (31) due to the supportive environment and accessible health services, which have been linked to improved academic performance and overall development (28, 32). As SBHCs are located within schools, they mitigate transportation issues and reduce the need for parents to miss a labor day to take their children to off-campus appointments (8, 10). This arrangement enhances parents' productivity while minimizing class absence for students (10). As a result, follow-up compliance with health care will likely improve, providing more consistent care for adolescents. Furthermore, health promotion within school efforts has a multiplier effect, where adolescents often share advice with peers and family, creating a network of healthier behaviors (2). This empowerment of adolescents to promote health is a significant benefit of SBHCs.

## Challenges and barriers to implementation

Despite the well-documented benefits of SBHCs, their implementation and expansion face significant barriers. One of the most pressing challenges is securing consistent funding. Many SBHCs rely on a combination of public funding, private grants, and insurance reimbursements, making financial stability uncertain. Studies indicate that SBHCs in low-income areas are particularly vulnerable to funding instability, affecting their long-term sustainability (8, 9). Additionally, there is a shortage of trained healthcare professionals willing to work in school-based settings, which can hinder service delivery. This is especially evident in rural regions, where attracting and retaining qualified staff remains a major challenge (10, 11). Disparities in access to SBHCs are also evident, with rural and low-income communities often lacking the resources to establish and sustain these centers.

Moreover, logistical and political challenges in implementing SBHCs in different countries, particularly in resource-limited settings, remain an obstacle. Infrastructure limitations, regulatory differences, and cultural perceptions of school-based healthcare significantly impact the feasibility of establishing and maintaining SBHCs in diverse regions (7, 31). Political will and public policy support also vary widely, influencing funding allocation and long-term sustainability. Addressing these obstacles requires coordinated efforts between governments, educational institutions, and healthcare systems to adapt SBHC models to diverse sociopolitical contexts. Ensuring success will demand comprehensive policy support, increased funding allocation, and initiatives to train and retain medical staff dedicated to adolescent health.

Beyond financial and workforce constraints, SBHCs also face challenges related to parental consent and concerns about their effectiveness compared to community-based health programs. Some regulatory frameworks require explicit parental consent for minors to access confidential health services, which can limit healthcare access for adolescents (11). Additionally, some argue that community-based health programs provide broader and more comprehensive services. However, rather than replacing these programs, SBHCs serve as a complementary approach that enhances accessibility and early intervention, particularly for underserved populations.

## Real-world impact of SBHCs

Several case studies illustrate the positive impact of SBHCs on adolescent health. For instance, a comprehensive review highlighted that SBHCs increase access to health services for children, families, and communities, leading to positive short- and long-term outcomes (10). Additionally, research indicates that SBHCs can significantly improve health outcomes for underserved youth by providing accessible and comprehensive services (32). Furthermore, during the Coronavirus Disease 2019 (COVID-19) pandemic, SBHCs faced challenges such as limited in-person access to students and increased severity of mental health issues. Despite these obstacles, many centers adapted by leveraging partnerships and community support to continue providing essential mental health services (33). However, further empirical evidence is needed to support claims that adolescents may prefer schools with SBHCs over those without.

## Conclusions

SBHCs enhance adolescent's health and overall development. It has been shown that SBHC cover a wide range of adolescent needs, from managing minor injuries, screening, and diagnosing of acute diseases or chronic conditions to providing preventive interventions that foster healthy lifestyles. However, to fully realize their potential, it is essential to address financial constraints, workforce shortages, and disparities in access. By prioritizing SBHCs in public health policies, governments can ensure equitable healthcare access for all adolescents.

Therefore, academia should continue producing rigorous, multidimensional, and comprehensive research as input for public health policy to evaluate, determine, and quantify the direct and indirect benefits of SHBCs in adolescents' health outcomes. This population is key to making interventions that promote healthy lifestyles and prevent chronic diseases in adulthood; such research is critical.

Based on the evidence, policymakers could appreciate and understand the power of SBHCs in adolescent health and wellbeing, leading them to prioritize SBHCs within primary health care.
